# Relationships between training load and body composition and physical fitness changes in sedentary individuals: A 4-month small-sided soccer games intervention

**DOI:** 10.1016/j.heliyon.2024.e27203

**Published:** 2024-02-29

**Authors:** Qi Xu, Rui Miguel Silva, Kai Qi, Dong Ma, TingYu Li, BaiQiao Pan, Filipe Manuel Clemente

**Affiliations:** a Gdansk University of Physical Education and Sport, 80-336, Gdańsk, Poland; b Sport Physical Activity and Health Research & Innovation Center (SPRINT), Viana do Castelo, Portugal; c Escola Superior Desporto e Lazer, Instituto Politécnico de Viana do Castelo, Rua Escola Industrial e Comercial de Nun’Álvares, 4900-347, Viana do Castelo, Portugal

**Keywords:** Football, Physical exercise, Health, Aerobic fitness

## Abstract

**Purpose:**

This study aimed to: (i) analyze the changes in physical fitness and body composition following a 4-month intervention of small-sided games (SSG) training; and (ii) analyze the association between internal and external training loads and the observed changes in physical fitness and body composition among sedentary young adults.

**Methods:**

Sixty sedentary individuals (males: 30; females: 30) participated in this randomized controlled trial study. Physical fitness and body composition parameters were assessed at the 1st, 8th weeks, and 16th weeks after a SSG intervention.

**Results:**

Significant main effects of time and gender on overall physical fitness parameters, with a notable time-group interaction were observed. For body composition measures, we found significant main effects of time, group, and gender. Furthermore, we identified significant correlations between shuttle run, handgrip, and vertical jump performance, and the time spent at VO2max (TVO2max) during SSG (r = −0.779, p = 0.001; r = −0.788, p = 0.001; r = 0.692, p = 0.004, respectively). Handgrip strength exhibited significant correlations with heat exhaustion (HE) and total distance (TD) during SSG (r = −0.616, p = 0.014; r = −0.629, p = 0.012). Similarly, we observed significant correlations between hip perimeter (HP), skinfolds (SF), waist-to-hip ratio (W:H), and TVO2max (r = 0.624, p = 0.013; r = 0.663, p = 0.007; r = 0.535, p = 0.040, respectively).

**Conclusion:**

This study indicates that the intensity achieved during SSG plays a crucial role in fostering positive adaptations in aerobic capacity, maximal strength, and jumping performance in recreational soccer. Therefore, practitioners should ensure that SSG formats generate the required stimulus to sustain prolonged periods within VO2max zones.

## 1 Introduction

Sedentary behavior is characterized as any waking activity with an energy expenditure of ≤1.5 metabolic equivalents (METs), while maintaining a seated, reclined, or lying posture [1]. Elevated levels of sedentary behavior have been correlated with an increased susceptibility to chronic diseases and adverse health outcomes [2]. It is crucial to distinguish between sedentary behavior and lack of physical activity, as the two concepts are distinct. Merely participating in physical activity does not inherently translate to a reduction in sedentary time [3].

Public health authorities advocate for adults to engage in either 150 min of moderate-intensity or 75 min of vigorous-intensity physical activity each week [4]. However, the approach to exercising can vary based on the chosen modality, and innovative, more time-efficient forms have emerged. For instance, analytical/traditional training approaches such as general strength and conditioning sessions can be less motivating than ecological approaches such as the practice of recreational team-based sports [5]. Within the context of sedentary populations, organized team-based training formats such as the use of small-sided games (SSG) have been increasing in interest [5]. The SSG offers a more enjoyable experience showing a positive influence on sedentary populations, effectively encouraging them to become more active participants [6].

The SSG is a small representation of soccer that modifies the structural dynamics of formal matches, which spans small formats (1v1 to 4v4), medium formats (5v5 to 8v8), and large formats (9v9 to 11v11). The examination of the relationships between internal load and external load with physical fitness changes is of paramount importance [7]. For instance, the cumulative rate of perceived exertion (RPE), as an internal load indicator, has been notably linked to substantial improvements in cardiorespiratory fitness [7]. Although SSG are proven to boost physical fitness in recreational groups, research on how SSG load affects fitness changes in sedentary young adults is still limited. Indeed, the predominant focus of the existing relationships has centered on professional soccer players rather than on untrained individuals [8].

Furthermore, considering individual variations in response to training loads, it is essential to determine the extent of these adaptations and their interaction with the physical stimuli provided by coaches during training sessions [9]. Based on the observed gaps in current research and the potential of SSG to influence physical fitness and body composition, this study hypothesizes that: (i) a 4-month intervention of SSG training will result in significant improvements in physical fitness and body composition among sedentary young adults; and (ii) there will be strong correlations between the internal and external training loads and the observed changes in physical fitness and body composition measures.

For all the reasons mentioned above, the aims of this study were two-fold: (i) to examine the physical fitness and body composition variations after a 4-month SSG training intervention, and (ii) to test the relationships between cumulative internal and external load measures and the changes in physical fitness and body composition measures within sedentary young adults.

## 2 Methods

### 2.1 Experimental design

**Fig. 1:**
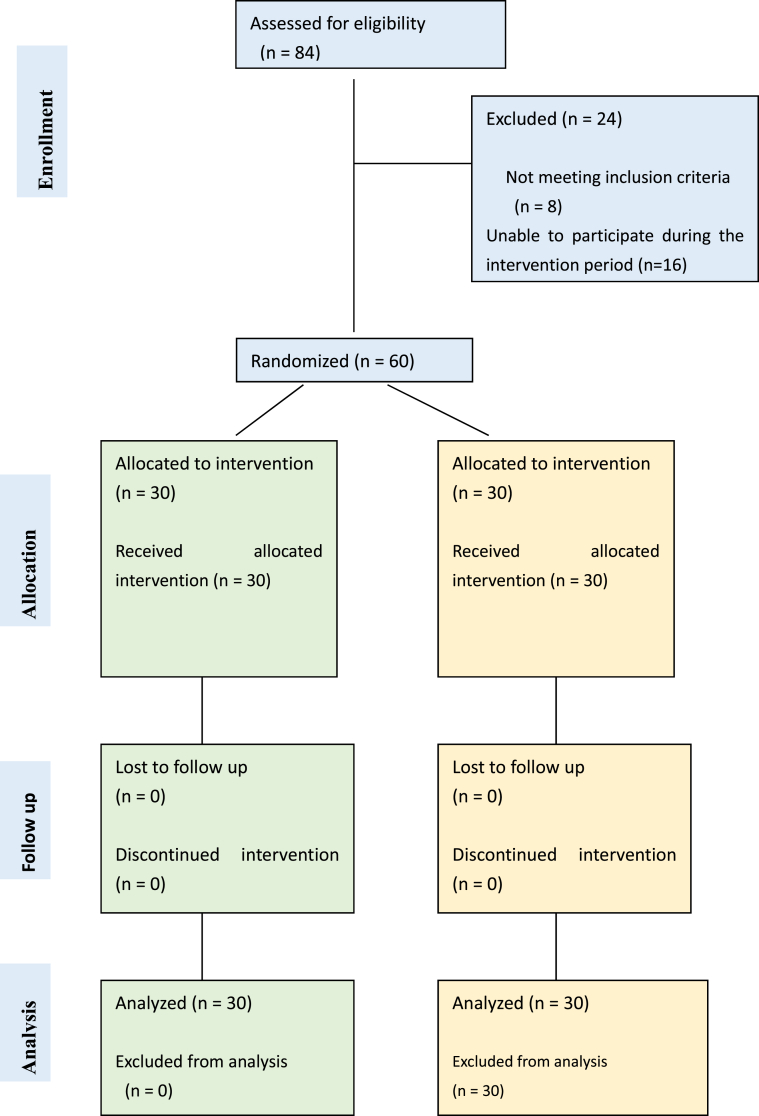
CONSORT flow diagram.

The present research adhered to the CONSORT guidelines for randomized controlled experiments (Fig. 1) [10]. This study employed a randomized controlled design, comprising two groups: one experimental group, which participated in SSGs, and one control group that continued their usual life routines. Assessments were conducted at three pivotal time points: baseline, midway through the intervention, and post-intervention. Participants were recruited through invitations extended to the university community of students, by email. Those who expressed interest were subsequently assessed for eligibility based on predetermined criteria. The individual responsible for determining the eligibility of each subject for inclusion in the trial was unaware of the subject's allocated group at the time of this decision.

Preliminary assessments of participants' physical fitness and analyses of their body composition were executed a week before the initiation of soccer SSGs training on January 6, 2023. After the initial assessments, the participants were randomly assigned to either the experimental or control group using a simple random sampling strategy. Letters were randomly assigned to the participants to determine their group. Subsequently, a second evaluation occurred in the eighth week of the baseline period, specifically on April 1, 2023. The examination took place on May 27, 2023, promptly following the conclusion of the training intervention. Uniform testing materials were utilized across all evaluations, albeit the testing environment exhibited fluctuations in temperature and relative humidity attributed to seasonal variations. The indoor track setting experienced temperature fluctuations ranging between 5 and 18 °C and relative humidity fluctuations between 47 and 55%. Spanning from January 12 to May 25, the training intervention spanned four months. Throughout this intervention phase, both external and internal loads were gauged utilizing global positioning and HR monitoring systems, respectively. Further data regarding the Rate of Perceived Exertion (RPE) were diligently recorded after each training session. It is noteworthy that the entire testing protocol and RPE data collection were meticulously administered by proficient personnel.

### 2.2 Participants

Using G*Power 3.1, we conducted an a priori sample size estimation based on a correlation point biserial model. With an effect size of 0.3 (considered moderate), a significance level of 0.05, and a power of 0.85, the analysis indicated that a total of 75 participants would be required for the study. To ensure robust statistical analysis and account for potential dropouts, 84 subjects were recruited. The study included sixty sedentary young adults, equally split between males and females, with 30 participants in both the experimental and control groups. The participants were, on average, 20.2 ± 1.0 years old, 1.67 ± 0.06 m tall, and weighed 86.3 ± 11.8 kg.

To ensure a diverse demographic, our study included participants from various universities who were sedentary, had a BMI over 28 and had no underlying health issues. They were required to adhere to the training program with over 85% compliance, refrain from professional sports training and drug-based weight loss strategies, and show no fatigue during assessments. Criteria for inclusion involved being minimally active and free from health conditions, while exclusion criteria prevented involvement in professional sports or drug-related weight loss methods. Detailed information regarding potential risks and discomfort was provided before participation, and written authorization was obtained from participants. The study received ethical approval from the Institutional Ethical Review Board at the Chengdu Institute of Physical Education, with reference code 2023#104.

### 2.3 Body composition

Body composition measurements were rigorously conducted between 7:30 a.m. and 8:00 a.m., before breakfast, to minimize potential dietary influences. Participants were explicitly instructed to refrain from any medications that might affect the assessment within 24 h before the measurements. The assessments were performed in triplicate within a controlled environment, maintaining temperatures between 5 and 18 °C Celsius for consistency. All data collection was carried out by a single experienced researcher to ensure uniformity and accuracy. Body mass was determined using a Smart Body Analyzer (WS-50, Withings, France) with a technical error margin of ±0.5 Kg. Skinfold thickness was measured meticulously using the Lange Skinfold Caliper (Beta Technology Incorporated, Cambridge, MD). To minimize data errors, all body measurements were taken three times, and the average was recorded as the final value. The Lange Skinfold Caliper (Beta Technology Incorporated, Cambridge, MD) has been validated against gold-standard methods, demonstrating strong correlations [11]. Changes in measurements between assessment moments were calculated to investigate their correlation with accumulated training load measures.

### 2.4 Fitness assessment

All assessments were completed on a single day, involving a sequence of tests. The testing protocol initiated with the grip strength assessment, followed by the standing broad jump and vertical jump evaluations. After an additional half-hour intermission, the 20-m shuttle run test was administered. Participants refrained from engaging in any strenuous physical activities the day before to ensure they were well-rested for the assessments. Moreover, participants were provided with prior information about the testing procedures to mitigate any potential biases in data collection.

### 2.5 20-M shuttle run test

In this test, participants were instructed to shuttle between two lines placed 20 m apart, including pivots around a central point positioned between these markers. Instead of stepping over the line when changing direction, participants had the option to pivot on the spot and continue running within the designated area. The test was concluded if a participant failed to reach a line in response to two consecutive beeps or ignored the beep signal twice in succession. The primary recorded parameter was the total distance covered by each participant. Participants were expected to exert their maximal effort during a single attempt. The estimation of VO2 max was calculated using the equation VO2max = −24.4 + 6.0 times the final velocity achieved. The changes in delta between assessment moments were specifically calculated to explore their relationships with the accumulated training load measures.

Given its progressive intensity, the 20-m shuttle run test aligns well with the fitness levels of our sedentary young adult participants, providing a safe and controlled environment for maximal effort exertion. Although is recognized the limitations of using a formula-based estimation of VO2 max, especially compared to direct measurement methods, the logistical and financial constraints, using a gold-standard method was out of the scope of this study.

### 2.6 Standing broad jump and vertical jump

Vertical jump height and standing broad jump horizontal distance in centimeters (cm) of the participants were assessed using the mobile application, My Jump2. The My Jump2 app, for jump measurements, has been validated against gold-standard measurements, showing acceptable validity and reliability [12]. Participants were instructed to assume a stance with their feet shoulder-width apart, hands placed on their hips, and knees flexed to a 90° angle. Subsequently, they were directed to generate maximal upward force in a vertical jump without employing their arms for propulsion. During the standing broad jump assessment, participants were asked to position themselves in front of a designated jumping line, with hands on their hips to prevent any arm movement while propelling forward. Each test was performed three times, and the most favorable result was selected for subsequent statistical analysis. A 3-min interval separated the two tests. The delta, representing the changes between assessment moments, was specifically calculated to explore their relationships with the accumulated training load measures.

### 2.7 Handgrip strength

Handgrip strength was assessed using the TKK dynamometer (TKK 5101 Grip-D, Takey, Tokyo, Japan), a device known for its minimal systematic error (0.49 kg) [13]. Participants received clear instructions to fully extend their arms and grasp the ergometer securely, exerting maximal force for a consistent 2-s duration, thus ensuring the precise measurement of grip strength. This assessment was repeated three times for both the left and right hands of each participant, with a 2-min rest interval between each trial. The measurements for the left and right hands were separated by a 3-min gap, and the highest grip strength value obtained was utilized for subsequent statistical analysis. The delta, signifying the changes between assessment moments, was specifically computed to explore their relationships with the accumulated training load measures.

### 2.8 Training load monitoring

#### 2.8.1 Internal load

Internal load measurements were meticulously documented in all sessions, employing a heart rate monitor (Polar RS400, Kempele, Finland) to accurately assess heart rate intensity. Each SSG intervention consisted of a minimum of one round and a maximum of three rounds. The maximum heart rate of participants was recorded during each rest period, and the highest maximum heart rate value was utilized as the statistical value. Concurrently, participants' levels of perceived exertion were carefully evaluated using the Borg CR10 scale. The objective assessment of internal load encompassed the recording of heart rate values at 20-min intervals during each training intervention, culminating in the collection of maximum heart rate data for statistical analysis. To ensure consistency and minimize device-related variations, each participant exclusively utilized the same heart rate monitor throughout all interventions.

Regarding subjective measures of internal load, participants were prompted to rate their perceived exertion (RPE) post-session, utilizing the Borg CR10 scale. Furthermore, participants were given the flexibility to assign an RPE score, ranging from 0 (indicating minimal exertion) to 10 (representing maximum exertion), to accurately capture their perceived effort. The duration of the sessions was also documented, and subsequently, the session-RPE was computed by multiplying the CR-10 Borg scale rating by the session duration in minutes [14]. For further data analysis, the total accumulated RPE and session-RPE between assessments, specifically between week 1 and week 8, and subsequently between week 9 and week 16, were computed. This facilitated an exploration of the association between perceived exertion and changes in body composition and physical fitness during these defined periods.

### 2.9 Training intervention and control group

Before the intervention commenced, all study participants received a 2-h lecture on the importance of physical activity and exercise in preventing non-communicable diseases, as well as guidance on adopting a healthy lifestyle, including tips for physical exercise and diet.

Participants assigned to the experimental group underwent five training sessions per week over a span of 16 weeks. Each session lasted between 10 and 20 min and included 1 to 3 exercises per session, utilizing small-sided games in 3v3 and 5v5 formats.

The control group maintained their daily routines and habits without any intervention from the research team. However, the research team remained available to offer recommendations and guidance to those interested in improving their physical activity and dietary habits. Nevertheless, the participants did not request structured training programs.

Both the control group and the experimental group participants were evaluated three times during the study period (prior to intervention, midway through intervention, and post-intervention) to assess their regular physical activity levels using the IPAQ short version questionnaire. This allowed for the monitoring of their physical activity habits.

Upon comparing pre-intervention to post-intervention data, it was observed that within the control group, 81% either maintained or decreased their physical activity levels. However, the remaining 19% experienced an increase in their moderate physical activity levels by 5–10%.

### 2.10 External load

Throughout all sessions, external load measurements were meticulously captured utilizing a portable 10Hz VX Motion SGPS unit (VX Motion, Wellington, New Zealand). The instrument's measurement methodology has been substantiated for its efficacy and reliability in data analysis, as evidenced by prior research [29]. Notably, this study integrates the parameter of TD as an integral facet of the external load measurement process. To facilitate further data analysis, the total accumulated distance covered between assessments (specifically, between week 1 and week 8, and subsequently between week 9 and week 16) was computed. This allowed for an association to be explored between the accumulated distance and the changes in body composition and physical fitness across these periods.

### 2.11 Statistical analysis

Tests of normal distribution and homogeneity (Kolmogorov–Smirnov and Levene's) were conducted on all data before analysis. A Mixed One-Way ANOVA (time x group) was conducted to test interactions between the three moments of repeated measures and the two groups. Partial eta squared (η^2^) was used to determine the effect size in the mixed ANOVA. To interpret the magnitude of the effect size, we adopted the following criteria [33]: η^2^ = 0.20, small; η^2^ = 0.50, medium; and η^2^ = 0.80, large. Posteriorly, the percentage change of physical fitness measures was calculated as follows: [100-(Pre*100)/Post]. Pearson's correlation coefficient r was used to examine the relationships between the accumulated training load measures of the first eight weeks of training with the physical fitness variations from the baseline to the eighth week of training. And between the accumulated training load measures of the last eight weeks of training with the physical fitness variations from the eighth week to the sixteenth week of training. To interpret the magnitude of these correlations, the following criteria were adopted [34]: r ≤ 0.1, trivial; 0.1 < r ≤ 0.3, small; 0.3 < r ≤ 0.5, moderate; 0.5 < r ≤ 0.7, large; 0.7 < r ≤ 0.9, very large; and r > 0.9, almost perfect. The statistical procedures were conducted on the statistical SPSS software (v25.0, IBM, Armonk, NY, USA) for a p < 0.05.

## 3 Results

The physical fitness performance assessments conducted on the first, eighth, and sixteenth weeks in control groups are displayed in Table 1.

**Table 1:** Descriptive statistics (Mean ± SD) of physical fitness assessments of Control Groups.

	Males			Females		
	1st Week	8th Week	16th Week	1st Week	8th Week	16th Week
**VO2max (ml/kg/min)**	26.5 ± 1.9	26.4 ± 1.7	26.7 ± 1.8	23.4 ± 1.7	23.6 ± 1.5	23.8 ± 1.4
**Handgrip Left (kg)**	37.4 ± 4.6	37.1 ± 5.2	37.0 ± 5.2	20.4 ± 4.0	20.9 ± 3.7	20.5 ± 3.2
**Handgrip Right (kg)**	40.0 ± 5.1	39.6 ± 5.5	39.1 ± 5.0	22.2 ± 4.3	22.1 ± 3.7	21.8 ± 3.2
**Vertical Jump (cm)**	19.6 ± 7.6	18.7 ± 5.6	19.0 ± 6.8	9.5 ± 1.6	9.3 ± 1.7	10.0 ± 2.0
**Horizontal Jump (cm)**	181.7 ± 14.4	181.5 ± 12.4	180.6 ± 11.6	163.9 ± 3.9	164.0 ± 3.8	164.74.5±

The physical fitness performance assessments conducted on the first, eighth, and sixteenth weeks in experimental groups are displayed in Table 2. The handgrip strength increased by 2.3% (left) and 3.1% (right) for females, and 1.8% (left) and 1.7% (right) for males. Horizontal jump improved by 14% for males and 33% for females, vertical jump increased by 2.3% for males and 4.1% for females, and VO2max improved by 6.5% for males and 10.1% for females. Body mass decreased by 9.7% (males) and 14.1% (females), BMI reduced by 10.7% (males) and 13.2% (females), waist circumference declined by 15.9% (males) and 14.1% (females), hip circumference decreased by 7.5% (males) and 9.5% (females), waist-to-hip ratios decreased by 2.4% (males) and 2.5% (females), and skinfolds decreased by 10.9% (males) and 19.7% (females) during the intervention period.

**Table 2:** Descriptive statistics (Mean ± SD) of physical fitness assessments of Experimental Groups.

	Males			Females		
	1st Week	8th Week	16th Week	1st Week	8th Week	16th Week
**VO2max (ml/kg/min)**	28.2 ± 3.2	30.3 ± 1.6	32.3 ± 1.0	23.0 ± 1.4	26.4 ± 1.5	29.1 ± 1.0
**Handgrip Left (kg)**	33.2 ± 4.4	33.6 ± 4.5	34.2 ± 4.7	23.1 ± 4.6	23.9 ± 4.5	24.4 ± 4.2
**Handgrip Right (kg)**	35.1 ± 4.4	35.9 ± 4.5	36.5 ± 4.8	25.3 ± 4.5	25.8 ± 4.3	26.6 ± 4.2
**Vertical Jump (cm)**	15.3 ± 4.7	18.5 ± 4.5	22.3.0 ± 5.0	9.0 ± 2.4	11.7 ± 2.9	15.5 ± 3.9
**Horizontal Jump (cm)**	173.3 ± 9.0	176.7 ± 9.0	181.8 ± 8.4	159.8 ± 6.8	162.8 ± 6.6	169.3 ± 4.8

For handgrip strength in the right arm, a significant main effect of time was observed (F = 2.525, p = 0.089, η^2^ = 0.084), with gender also exhibiting a significant main effect (F = 142.277, p < 0.001, η^2^ = 0.718). A highly significant interaction effect between time and group was observed (F = 20.107, p < 0.001, η^2^ = 0.422). The left arm's handgrip strength revealed a significant main effect of time (F = 7.609, p < 0.001, η^2^ = 0.120), along with a significant main effect of gender (F = 136.093, p < 0.001, η^2^ = 0.708). However, the group factor displayed no significant main effect (F = 0.011, p = 0.915, η^2^ = 0.000). In the case of the horizontal jump, a significant main effect of time was observed (F = 61.418, p < 0.001, η^2^ = 0.523), with gender also showing a significant main effect (F = 48.785, p < 0.001, η^2^ = 0.466). Nevertheless, the group factor had no significant main effect (F = 0.946, p = 0.335, η^2^ = 0.017). For the vertical jump, a significant main effect of time was detected (F = 47.426, p < 0.001, η^2^ = 0.459), along with a significant main effect of gender (F = 55.368, p < 0.001, η^2^ = 0.497). However, the group factor did not exhibit a significant main effect (F = 0.907, p = 0.345, η^2^ = 0.016). Concerning VO2max, a significant main effect of time was found (F = 83.012, p < 0.001, η^2^ = 0.597), along with a significant main effect of gender (F = 83.486, p < 0.001, η^2^ = 0.599). The group factor displayed a significant main effect (F = 67.010, p < 0.001, η^2^ = 0.545).

Another mixed ANOVA was conducted to evaluate body composition measures. Body mass exhibited a significant main effect of time (F = 115.781, p < 0.001, η^2^ = 0.805), with gender also demonstrating a significant main effect (F = 6.162, p = 0.019, η^2^ = 0.180). The group factor had a significant main effect (F = 3.396, p = 0.076, η^2^ = 0.108). For BMI, a significant main effect of time was noted (F = 82.450, p < 0.001, η^2^ = 0.746), and the group factor displayed a statistically significant main effect (F = 5.695, p = 0.024, η^2^ = 0.169). Waist circumference revealed a significant main effect of time (F = 149.971, p < 0.001, η^2^ = 0.843), along with a significant main effect of gender (F = 8,048, p = 0.008, η^2^ = 0.223). The group factor also exhibited a significant main effect (F = 9.462, p = 0.005, η^2^ = 0.253). Hip circumference displayed a significant main effect of time (F = 122.670, p < 0.001, η^2^ = 0.814), along with a significant main effect of gender (F = 9.484, p = 0.005, η^2^ = 0.253). The group factor had a significant main effect (F = 8.645, p = 0.007, η^2^ = 0.236). Waist-to-hip ratios indicated a significant main effect of time (F = 15.027, p < 0.001, η^2^ = 0.349), while no significant effects were observed for gender or the group factor. Finally, skinfolds showed a significant main effect of time (F = 125.813, p < 0.001, η^2^ = 0.818), and the group factor displayed a significant main effect (F = 7.624, p = 0.010, η^2^ = 0.214), though gender did not exhibit a significant main effect.

A correlation analysis was conducted between the accumulated training load measures of the first eight weeks of training with the physical fitness performance variations from the baseline to the eighth week of training (Table 3). And between the accumulated training load measures of the last eight weeks of training with the physical fitness variations from the eighth week to the sixteenth week of training (Table 4).

**Table 3:** Correlations between the training load from the 1st to the 8th weeks, and the physical fitness variation from baseline to the eighth week.

Males	s-RPE (A.U.)	HRmax (Bpm)	T_VO2max_ (min.)	AO (%)	HE(kcal)	TD (m)
**Shuttle run (mL/(kg·min)**	*r* = −0.312; *p* = 0.255	*r* = 0.465 *p* = 0.080	*r* = −0.779 *p* = 0.001**	*r* = 0.304 *p* = 0.271	*r* = 0.057 *p* = 0.841	*r* = 0.006 *p* = 0.984
**HL (kg)**	*r* = −0.085; *p* = 0.764	*r* = 0.120 *p* = 0.670	*r* = −0.210 *p* = 0.452	*r* = −0.046 *p* = 0.872	*r* = 0.246 *p* = 0.376	*r* = 0.240 *p* = 0.389
**HR (kg)**	*r* = −0.483 *p* = 0.068	*r* = 0.264 *p* = 0.342	*r* = −0.495 *p* = 0.061	*r* = 0.198 *p* = 0.479	*r* = −0.088 *p* = 0.756	*r* = −0.136 *p* = 0.630
**HJ (cm)**	*r* = 0.180 *p* = 0.520	*r* = 0.046 *p* = 0.872	*r* = −0.002 *p* = 0.995	*r* = −0.241 *p* = 0.386	*r* = 0.402 *p* = 0.138	*r* = 0.369 *p* = 0.176
**VJ (cm)**	*r* = 0.014 *p* = 0.961	*r* = 0.092 *p* = 0.744x	*r* = −0.135 *p* = 0.631	*r* = −0.342 *p* = 0.212	*r* = 0.269 *p* = 0.333	*r* = 0.216 *p* = 0.439
**Females**	**s-RPE (A.U.)**	**HRmax (Bpm)**	**T**_**VO2max**_**(min.)**	**AO (%)**	**HE(kcal)**	**TD (m)**
**Shuttle run (mL/(kg·min)**	*r* = 0.029 *p* = 0.919	*r* = −0.156 *p* = 0.579	*r* = −0.372 *p* = 0.172	*r* = −0.130 *p* = 0.645	*r* = 0.138 *p* = 0.624	*r* = 0.127 *p* = 0.652
**HL (kg)**	*r* = 0.319 *p* = 0.247	*r* = −0.159 *p* = 0.572	*r* = −0.372 *p* = 0.172	*r* = −0.312 *p* = 0.172	*r* = −0.095 *p* = 0.736	*r* = −0.019 *p* = 0.946
**HR (kg)**	*r* = −0.367 *p* = 0.178	*r* = 0.264 *p* = 0.341	*r* = −0.788 *p* = 0.001**	*r* = −0.414 *p* = 0.125	*r* = 0.262 *p* = 0.345	*r* = 0.265 *p* = 0.340
**HJ (cm)**	*r* = −0.034 *p* = 0.905	*r* = 0.364 *p* = 0.182	*r* = −0.034 *p* = 0.904	*r* = −0.233 *p* = 0.403	*r* = −0.197 *p* = 0.481	*r* = −0.227 *p* = 0.415
**VJ (cm)**	*r* = −0.111 *p* = 0.693	*r* = 0.401 *p* = 0.138	*r* = −0.159 *p* = 0.571	*r* = −0.259 *p* = 0.352	*r* = 0.156 *p* = 0.578	*r* = 0.116 *p* = 0.681

A.U.: arbitrary units; HL: handgrip left arm; HR: handgrip right arm; HJ: horizontal jump; VJ: vertical jump; s-RPE: session-rate of perceived exertion; AO: arterial oxygen; HE: heat exhaustion; TD: total distance; T_VO2max:_ time spent in VO2max during exercise.

**Table 4:** Correlations between the training load from the 8th to the 16th weeks, and the physical fitness measures assessed at the sixteenth week.

Males	s-RPE (A.U.)	HRmax (Bpm)	T_VO2max_ (min.)	AO (%)	HE(kcal)	TD (m)
**Shuttle run (mL/(kg·min)**	*r* = −0.067 *p* = 0.811	*r* = 0.162 *p* = 0.563	*r* = −0.314 *p* = 0.254	*r* = 0.182 *p* = 0.515	*r* = 0.269 *p* = 0.332	*r* = 0.274 *p* = 0.323
**HL (kg)**	*r* = −0.045 *p* = 0.874	*r* = 0.094 *p* = 740	*r* = 0.0.006 *p* = 0.984	*r* = 0.500 *p* = 0.058	*r* = 0.102 *p* = 0.718	*r* = 0.099 *p* = 0.726
**HR (kg)**	*r* = −0.268 *p* = 0.333	*r* = 0.226 *p* = 0.418	*r* = −0.303 *p* = 0.273	*r* = 0.107 *p* = 0.705	*r* = 0.020 *p* = 0.944	*r* = 0.018 *p* = 0.948
**HJ (cm)**	*r* = 0.040 *p* = 0.887	*r* = −0.083 *p* = 0.769	*r* = 0.-0.311 *p* = 0.259	*r* = 0.110 *p* = 0.696	*r* = −0.226 *p* = 0.417	*r* = −0.243 *p* = 0.383
**VJ (cm)**	*r* = 0.010 *p* = 0.972	*r* = 0.051 *p* = 0.856	*r* = 0.692 *p* = 0.004**	*r* = −0.151 *p* = 0.592	*r* = −0.295 *p* = 0.286	*r* = −0.319 *p* = 0.246
**Females**	**s-RPE (A.U.)**	**HRmax (Bpm)**	**T**_**VO2max**_**(min.)**	**AO (%)**	**HE(kcal)**	**TD (m)**
**Shuttle run (mL/(kg·min)**	*r* = 0.122 *p* = 0.655	*r* = 0.172 *p* = 0.539	*r* = −0.428 *p* = 0.112	*r* = −0.255 *p* = 0.360	*r* = −0.034 *p* = 0.904	*r* = −0.114 *p* = 0.686
**HL (kg)**	*r* = 0.045 *p* = 0.874	*r* = 0.234 *p* = 0.402	*r* = −0.127 *p* = 0.652	*r* = 0.078 *p* = 0.781	*r* = 0.240 *p* = 0.390	*r* = 0.250 *p* = 0.368
**HR (kg)**	*r* = 0.018 *p* = 0.948	*r* = 0.161 *p* = 0.567	*r* = −0.186 *p* = 0.508	*r* = 0.424 *p* = 0.115	*r* = −0.616 *p* = 0.014**	*r* = −0.629 *p* = 0.012**
**HJ (cm)**	*r* = 0.284 *p* = 0.306	*r* = 0.243 *p* = 0.382	*r* = −0.099 *p* = 0.726	*r* = −0.008 *p* = 0.978	*r* = −0.243 *p* = 0.3830	*r* = −0.254 *p* = 0.361
**VJ (cm)**	*r* = −0.295 *p* = 0.285	*r* = −0.260 *p* = 0.349	*r* = 0.575 *p* = 0.025**	*r* = −0.312 *p* = 0.257	*r* = 0.223 *p* = 0.424	*r* = 0.210 *p* = 0.452

A.U.: arbitrary units; HL: handgrip left arm; HR: handgrip right arm; HJ: horizontal jump; VJ: vertical jump; s-RPE: session-rate of perceived exertion; AO: arterial oxygen; HE: heat exhaustion; TD: total distance; T_VO2max:_ time spent in VO2max during exercise.

A new correlation analysis was conducted between the accumulated training load measures of the first eight weeks of training with the body composition measures variations from the baseline to the eighth week of training (Table 5). Between the accumulated training load measures of the last eight weeks of training with the body composition measures variations from the eighth week to the sixteenth week of training (Table 6).

**Table 5:** Correlations between the training load from the 1st to the 8th weeks, and the body composition variations from baseline to the eighth week.

Males	s-RPE (A.U.)	HRmax (Bpm)	T_VO2max_ (min.)	AO (%)	HE(kcal)	TD (m)
**BM (kg)**	*r* = 0.443 *p* = 0.098	*r* = −0.042 *p* = 0.881	*r* = 0.442 *p* = 0.099	*r* = −0.156 *p* = 0.578	*r* = 0.324 *p* = 0.239	*r* = 0.321 *p* = 0.243
**BMI (kg/m**^**2**^**)**	*r* = 0.194 *p* = 0.489	*r* = −0.248 *p* = 0.373	*r* = 0.077 *p* = 0.785	*r* = −0.161 *p* = 0.568	*r* = 0.374 *p* = 0.169	*r* = 0.311 *p* = 0.260
**WC (cm)**	*r* = 0.141 *p* = 0.615	*r* = −0.369 *p* = 0.176	*r* = 0.415 *p* = 0.124	*r* = −0.432 *p* = 0.108	*r* = 0.224 *p* = 0.422	*r* = 0.210 *p* = 0.452
**HP (cm)**	*r* = 0.379 *p* = 0.164	*r* = −0.622 *p* = 0.013	*r* = 0.624 *p* = 0.013*	*r* = −0.226 *p* = 0.417	*r* = −0.135 *p* = 0.631	*r* = −0.097 *p* = 0.730
**W:H (%)**	*r* = −0.288 *p* = 0.298	*r* = 0.482 *p* = 0.069	*r* = −0.499 *p* = 0.058	*r* = 0.127 *p* = 0.652	*r* = 0.220 *p* = 0.432	*r* = 0.170 *p* = 0.544
**SF (mm)**	*r* = 0.601 *p* = 0.018*	*r* = −0.425 *p* = 0.114	*r* = 0.663 *p* = 0.007**	*r* = 0.080 *p* = 0.776	*r* = 0.293 *p* = 0.290	*r* = 0.328 *p* = 0.232
**Females**	**s-RPE (A.U.)**	**HRmax (Bpm)**	**T**_**VO2max**_**(min.)**	**AO (%)**	**HE(kcal)**	**TD (m)**
**BM (kg)**	*r* = −0.456 *p* = 0.088	*r* = −0.091 *p* = 0.747	*r* = −0.105 *p* = 0.710	*r* = 0.421 *p* = 0.119	*r* = 0.062 *p* = 0.827	*r* = 0.122 *p* = 0.665
**BMI (kg/m**^**2**^**)**	*r* = −0.067 *p* = 0.812	*r* = −0.143 *p* = 0.610	*r* = 0.173 *p* = 0.537	*r* = 0.244 *p* = 0.381	*r* = −0.094 *p* = 0.738	*r* = −0.099 *p* = 0.727
**WC (cm)**	*r* = −0.291 *p* = 0.293	*r* = −0.021 *p* = 0.942	*r* = 0.060 *p* = 0.831	*r* = 0.697 *p* = 0.004**	*r* = 0.071 *p* = 0.802	*r* = 0.115 *p* = 0.682
**HP (cm)**	*r* = −0.439 *p* = 0.102	*r* = −0.149 *p* = 0.595	*r* = −0.129 *p* = 0.648	*r* = 0.333 *p* = 0.225	*r* = −0.037 *p* = 0.896	*r* = 0.017 *p* = 0.953
**W:H (%)**	*r* = 0.389 *p* = 0.152	*r* = 0.079 *p* = 0.779	*r* = 0.535 *p* = 0.040*	*r* = 0.135 *p* = 0.631	*r* = −0.155 *p* = 0.580	*r* = −0.161 *p* = 0.567
**SF (mm)**	*r* = −0.356 *p* = 0.192	*r* = 0.070 *p* = 0.803	*r* = −0.077 *p* = 0.784	*r* = 0.245 *p* = 0.380	*r* = −0.094 *p* = 0.738	*r* = −0.051 *p* = 0.858

A.U.: arbitrary units; BM: body mass; BMI: body mass index; WC: waist circumference; HP: hip perimeter; W:H: waist-to-hip ratio; SF: skinfold thickness; s-RPE: session-rate of perceived exertion; AO: arterial oxygen; HE: heat exhaustion; TD: total distance; T_VO2max:_ time spent in VO2max during exercise.

**Table 6:** Correlations between the training load from the 8th to the 16th weeks, and the body composition variations from the eighth to the sixteenth week.

Males	s-RPE (A.U.)	HRmax (Bpm)	T_VO2max_ (min.)	AO (%)	HE(kcal)	TD (m)
**BM (kg)**	*r* = 0.039 *p* = 0.889	*r* = −0.356 *p* = 0.193	*r* = 0.330 *p* = 0.230	*r* = −0.323 *p* = 0.241	*r* = 0.280 *p* = 0.312	*r* = 0.292 *p* = 0.290
**BMI (kg/m**^**2**^**)**	*r* = 0.389 *p* = 0.152	*r* = −0.068 *p* = 0.809	*r* = −0.037 *p* = 0.895	*r* = 0.256 *p* = 0.358	*r* = −0.245 *p* = 0.379	*r* = −0.208 *p* = 0.457
**WC (cm)**	*r* = −0.009 *p* = 0.976	*r* = −0.376 *p* = 0.167	*r* = 0.036 *p* = 0.900	*r* = −0.296 *p* = 0.284	*r* = 0.120 *p* = 0.670	*r* = 0.102 *p* = 0.719
**HP (cm)**	*r* = −0.056 *p* = 0.842	*r* = −0.171 *p* = 0.543	*r* = 0.369 *p* = 0.176	*r* = 0.085 *p* = 0.764	*r* = 0.252 *p* = 0.365	*r* = 0.261 *p* = 0.347
**W:H (%)**	*r* = −0.054 *p* = 0.849	*r* = −0,519 *p* = 0.048*	*r* = −0.183 *p* = 0.514	*r* = −0.558 *p* = 0.031*	*r* = −0.048 *p* = 0.865	*r* = −0.088 *p* = 0.755
**SF (mm)**	*r* = 0.045 *p* = 0.873	*r* = −0.221 *p* = 0.429	*r* = 0.443 *p* = 0.098	*r* = 0.239 *p* = 0.390	*r* = 0.379 *p* = 0.163	*r* = 0.374 *p* = 0.169
**Females**	**s-RPE (A.U.)**	**HRmax (Bpm)**	**T**_**VO2max**_**(min.)**	**AO (%)**	**HE(kcal)**	**TD (m)**
**BM (kg)**	*r* = 0.182 *p* = 0.517	*r* = 0.061 *p* = 0.830	*r* = −0.079 *p* = 0.781	*r* = 0.090 *p* = 0.749	*r* = −0.287 *p* = 0.299	*r* = −0.350 *p* = 0.201
**BMI (kg/m**^**2**^**)**	*r* = −0.064 *p* = 0.821	*r* = −0.007 *p* = 0.981	*r* = −0.147 *p* = 0.601	*r* = 0.141 *p* = 0.616	*r* = −0.389 *p* = 0.152	*r* = −0.405 *p* = 0.134
**WC (cm)**	*r* = −0.186 *p* = 0.508	*r* = −0.230 *p* = 0.410	*r* = −0.086 *p* = 0.759	*r* = −0.130 *p* = 0.645	*r* = 0.082 *p* = 0.770	*r* = 0.132 *p* = 0.639
**HP (cm)**	*r* = −0.115 *p* = 0.682	*r* = 0.217 *p* = 0.438	*r* = −0.434 *p* = 0.106	*r* = −0.105 *p* = 0.710	*r* = −0.074 *p* = 0.792	*r* = −0.144 *p* = 0.609
**W:H (%)**	*r* = 0.068 *p* = 0.810	*r* = −0.543 *p* = 0.037*	*r* = 0.423 *p* = 0.116	*r* = 0.062 *p* = 0.825	*r* = 0.166 *p* = 0.554	*r* = 0.287 *p* = 0.300
**SF (mm)**	*r* = 0.083 *p* = 0.768	*r* = 0.411 *p* = 0.128	*r* = -,084 *p* = 0.767	*r* = ,050 *p* = 0.860	*r* = −0.093 *p* = 0.741	*r* = −0.208 *p* = 0.456

A.U.: arbitrary units; BM: body mass; BMI: body mass index; WC: waist circumference; HP: hip perimeter; W:H: waist-to-hip ratio; SF: skinfold thickness; s-RPE: session-rate of perceived exertion; AO: arterial oxygen; HE: heat exhaustion; TD: total distance; T_VO2max:_ time spent in VO2max during exercise.

## 4 Discussion

This study investigated the impact of a 16-week small-sided games intervention on sedentary individuals. The findings revealed significant improvements in handgrip strength, horizontal and vertical jump performance, and VO2 max between the eighth and 16th week. Significant changes in overall body composition measures among the experimental groups were found. Significant correlations were observed between exercise duration and improvements in VO2 max, HRmax, and vertical jump. Additionally, associations were found between handgrip strength, total distance, and heat exhaustion. While, body composition changes were associated with HRmax, VO2max, and arterial oxygen consumption during exercise.

Both vertical and horizontal jump performance significantly improved with SSG interventions. VO2max substantially increased with SSG interventions, with the group factor significantly influencing VO2max, emphasizing the effectiveness of the experimental group's training regimen in enhancing aerobic capacity. Indeed, a systematic review that summarized the evidence on the acute and long-term effects of SSG on the physical fitness of untrained individuals revealed that SSG interventions had positive effects on physical fitness, including VO2 max [8].

Handgrip strength in both arms exhibited time-dependent improvement influenced by gender disparities, aligning with established upper body strength norms [15]. A previous study that investigated the impact of a 12-week recreational football intervention on the physical fitness of sedentary individuals showed no significant differences in handgrip strength [16]. However, the strength improvements observed in the present study may be attributed to the dynamic nature of SSG, which involves frequent high-intensity bursts of activity, comprised of changes of direction, and jumping, among other movements, that declare the need to show good strength levels [17]. Given that handgrip strength has been closely linked to overall muscle strength, with correlation coefficients ranging from 0.736 to 0.890, this fact could account for the observed improvements in handgrip strength [18]. Finally, the group factor significantly impacted all body composition measures, indicating more pronounced alterations in the experimental group, possibly reflecting enhanced overall health and fitness after the SSG intervention [8]. Although these observations may be attributed to the specific intervention method employed, further investigations are necessary to discern the precise mechanisms behind these improvements.

Considering the relationships found between training load during the SSGs and the physical fitness changes, this study reported interesting evidence. Specifically, the higher s-RPE values resulted in improved time spent at VO2max. Therefore, the training intensity perceived during SSGs plays a pivotal role in driving cardiovascular fitness improvements. This finding aligns with the existing body of research emphasizing the significance of high-intensity training sessions in enhancing the capacity to tolerate more time at or near VO2 max during exercise [[19], [20], [21]]. Interestingly, handgrip strength correlated with the time spent at VO2max and shuttle run performance. This finding has been supported by prior studies examining the connection between aerobic exercise and handgrip strength. These studies have shown that sedentary individuals who engage in less aerobic exercise are at a higher risk of experiencing reduced handgrip strength [22,23].

Significant correlations were reported Regarding the relationships between training load and anthropometry measures. For instance, the time spent at VO2max displayed significant correlations with skinfolds, waist circumference, and waist-to-hip ratio. These findings provide valuable insights into the intricate relationships between training load during SSGs and body composition adaptations. Such findings may be explained by increases in soccer training volume and intensity. This evidence is supported by previous findings that confirmed that changes in body composition are strongly influenced by the intensity of training and the duration of the intervention [24].

Similarly, a systematic review identified the training effects of recreational soccer (which included SSGs) on overall health in untrained individuals [25]. Specifically, the authors found that both males and females experienced a significant reduction in body fat mass and increases in lean body mass as a consequence of a recreational soccer intervention [25]. Despite some indicators were found to be correlated, most indicators did not show a significant correlation. This suggests that adaptations are not exclusively attributable to the training load parameters investigated.

This study has some limitations that warrant consideration. The small sample size employed herein raises concerns regarding the broader applicability of the results. Although we carefully structured the sequence of tests from least to most physically demanding, aiming to minimize fatigue and ensure reliable performance across tests, it must be recognizable the lack of randomization in controlling for the testing order sequence effects as a potential limitation. Furthermore, the exclusive inclusion of students precludes a comprehensive representation of diverse social groups, potentially compromising the generalizability of our findings. Longitudinal studies tracking these adaptations over more extended periods could help establish the long-term sustainability of the observed improvements, offering practical implications for exercise prescription and health promotion.

## 5 Conclusion

The 4-month intervention involving SSG in soccer led to significant improvements in physical fitness and body composition among sedentary young adults. These findings highlight the efficacy of structured SSG programs in enhancing fitness levels. The significant correlations between training load measures during the SSG intervention and changes in physical fitness and body composition over time underscore the importance of monitoring load to track performance adaptations in sedentary individuals. Furthermore, the ability of these games to keep participants within VO2max zones for prolonged periods is crucial for promoting improvements in aerobic capacity, strength, and jumping performance. However, despite the observed correlations, caution is warranted in acknowledging the potential presence of other confounding variables, emphasizing the necessity for further extensive research in this field.

## Ethics statement

Detailed information regarding potential risks and discomfort was provided before participation, and written authorization was obtained from participants. The study received ethical approval from the Institutional Ethical Review Board at the Chengdu Institute of Physical Education, with reference code 2023#104.

## Informed consent

Informed consent was obtained from all patients to participate in the study and have their data published in a scientific article under the condition of remaining anonymous. No personal information is disclosed in the publication.

## CRediT authorship contribution statement

**Qi Xu:** Writing – review & editing, Writing – original draft, Methodology, Investigation, Data curation, Conceptualization. **Rui Miguel Silva:** Writing – review & editing, Writing – original draft, Formal analysis. **Kai Qi:** Writing – review & editing, Writing – original draft. **Dong Ma:** Writing – review & editing, Writing – original draft. **TingYu Li:** Writing – review & editing, Writing – original draft. **BaiQiao Pan:** Writing – review & editing, Writing – original draft. **Filipe Manuel Clemente:** Writing – review & editing, Writing – original draft, Supervision, Methodology, Conceptualization.

## Declaration of competing interest

The authors declare that they have no known competing financial interests or personal relationships that could have appeared to influence the work reported in this paper.
